# Antimicrobial resistance (AMR) as a form of human–wildlife conflict: Why and how nondomesticated species should be incorporated into AMR guidance

**DOI:** 10.1002/ece3.10421

**Published:** 2023-08-31

**Authors:** Jessica Mitchell

**Affiliations:** ^1^ Nuffield Centre for International Health and Development, Leeds Institute for Health Sciences, Faculty of Medicine and Health University of Leeds Leeds UK

**Keywords:** antimicrobial resistance, human–wildlife conflict, one health, wildlife

## Abstract

The challenge of antimicrobial resistance (AMR) continues to receive significant global attention as common infections become increasingly resistant to the drugs used to treat them. Once an infectious microbe has developed a mechanism of resistance, it can cause longer, more damaging infections which are more costly, time‐consuming, and sometimes impossible to treat. Such impacts occur across the health of humans, animals, plants, and the environment. Thus, AMR is considered a One Health issue. However, current narratives on AMR focus on humans, food‐producing animals, crops, and their immediate environments. Very little attention is given to wildlife in terms of the impact of AMR on their health, nor their role in the evolution and spread of AMR. This article (1) discusses an absence of wildlife in current AMR guidance, (2) suggests how this absence of wildlife could limit understanding of, and action on, AMR, (3) proposes that considering AMR as a form of human–wildlife conflict could enable AMR guidance to better incorporate wildlife into action planning and create a truly One Health approach to tackle AMR.

## 1 BACKGROUND

Antimicrobial resistance (AMR) threatens human, animal, and environmental health (O'Neill, 2016; WHO, 2015). AMR naturally occurs when microbes attempt to endure threats to their survival. Because microbes have short generation times and multiple ways of sharing their genetic information, any mechanism they find to evolve resistance can spread rapidly. A common driver of AMR is the so‐called *misuse* of antimicrobials, which tends to refer to situations whereby the wrong drug is used to treat an infection; the correct medication is used but in too small or short a dosage, or when antimicrobials are used as prophylaxis to prevent future infections or as growth promotors (Batista et al., 2020; Holmes, Moore, et al., 2016; Van Boeckel et al., 2015; Vikesland et al., 2019). Antimicrobial *misuse* can be convenient in the short term but drives AMR by exposing microbes to nonlethal doses of medication, which can prompt them to evolve mechanisms of resistance. Because AMR knows no geographic or species boundaries, such resistant microbes can then move between human and animal bodies and the soil, water, air, and plants within our environments. AMR is thus described as a One Health or Planetary Health challenge (Bresalier et al., 2015; CDCP, 2017).

However, the majority of AMR research, practice, and guidance focuses on humans, domestic animals such as livestock and pets, crops, and the environments in which these species live (Auta et al., 2019; Cavicchioli et al., 2019; Holmes, Moore, et al., 2016; Reddy et al., 2022; Van Boeckel et al., 2015; Vikesland et al., 2019). Wildlife and habitats beyond those directly linked to people and livestock are rarely considered within AMR surveillance programs (Arnold et al., 2016), while the impacts of AMR upon the health of wildlife and their environments are to the best of our knowledge absent from the literature. This is concerning given that wildlife is a key component of life on Earth and intersects with the expanding human population more frequently than ever (Bresalier et al., 2015; Otu et al., 2021; Shaheen, 2022). Wildlife and nature‐based interactions are also recognized to have positive impacts on the physical and mental health of humans (Brymer et al., 2019; Dobson et al., 2021; Dunkley, 2023; Pooley et al., 2021). Thus, safeguarding wildlife from AMR is likely to have a multitude of One Health benefits, including the protection of valuable connections between humans and nondomesticated species.

It is not just antimicrobial (mis)use, which drives AMR; heavy metal and agro‐chemical/pesticide (mis)use, temperature, humidity, and radiation changes are all recognized to challenge the same mobile genetic elements, which drive resistance in microbes (Cavicchioli et al., 2019; Holmes, Moore, et al., 2016). Wildlife and the infectious microbes they host are frequently exposed to such stressors due to climate change, pollution, and habitat loss (Bresalier et al., 2015; Kutz et al., 2009; Otu et al., 2021; Swift et al., 2019). Many of these interactions result from or can develop into conflict between humans and wildlife for access to resources including habitats, water, territories, and food, but they can also lead to the spillover of diseases between wild, domesticated, and human populations (Cui et al., 2023; Greig et al., 2015; Skarżyńska et al., 2021). Thus, lack of engagement with AMR drivers in wildlife could thus mean we are missing key developments in microbial evolution, transmission pathways for the (re)emergence of infectious pathogens, and hidden impacts of human–wildlife conflict (HWC).

This article reflects on the limited discussion of wildlife within current AMR narratives and considers routes to incorporating wildlife as an important component in the understanding and prevention of AMR. We propose the argument that AMR could be considered an example of HWC because many of its One Health drivers are exacerbated by resource exploitation, ecosystem damage and pollution. Finally, we suggest how AMR documentation could utilize existing HWC guidance to enhance One Health narratives around AMR, specifically by considering wider data sources, stakeholder engagement, and action planning that relates to nondomesticated animals and environments.

## 2 THE ABSENCE OF WILDLIFE AND WILD SPACES IN CURRENT AMR GUIDANCE

There is an absence of wildlife within National Policy and Global level guidance on AMR action. This is both in terms of discussing AMR surveillance in nonhuman and nonagricultural settings and in terms of the impacts of AMR on the health of nonhuman and nondomestical animals. A recent machine learning and topic modeling analysis of global peer‐reviewed literature on AMR could not identify wildlife‐associated topics within the 158,616 articles screened (Luz et al., 2022). Additionally, an exploratory rapid screening of gray literature conducted between November 2022 and January 2023 revealed that the term wildlife did not appear in a subset Global level AMR guidance such as WHO and Wellcome Trust reports (see Table S1). Wildlife was not considered in country level National AMR Action Plans which are WHO‐supported documents to guide AMR action at country level (WHO, 2015). Even the World Organization for Animal Health (WOAH, formally known as OIE) do not specifically mention wildlife in their AMR materials, while AMR is not addressed within their 2023 report “Wildlife health surveillance: gaps, needs and opportunities” (Delgado et al., 2023). When looking at AMR and One Health documentation at country‐specific level, Ghana provides some incorporation of nondomesticated animals in their Zoonosis and pandemic preparedness (Strengthening Pandemic Preparedness in Ghana, 2022) and climate‐smart agriculture plans (Bank W, 2020), which in turn reference their National AMR Action Plan (WHO, 2015). This is a promising example of how linkages between wildlife health and surveillance can be made to AMR. However, overall, there is very limited consideration of wildlife and nondomestical animals in any AMR documentation or guidance.

## 3 EXCLUDING WILDLIFE COULD LIMIT UNDERSTANDING OF AND ACTION ON AMR

**FIGURE 1:**
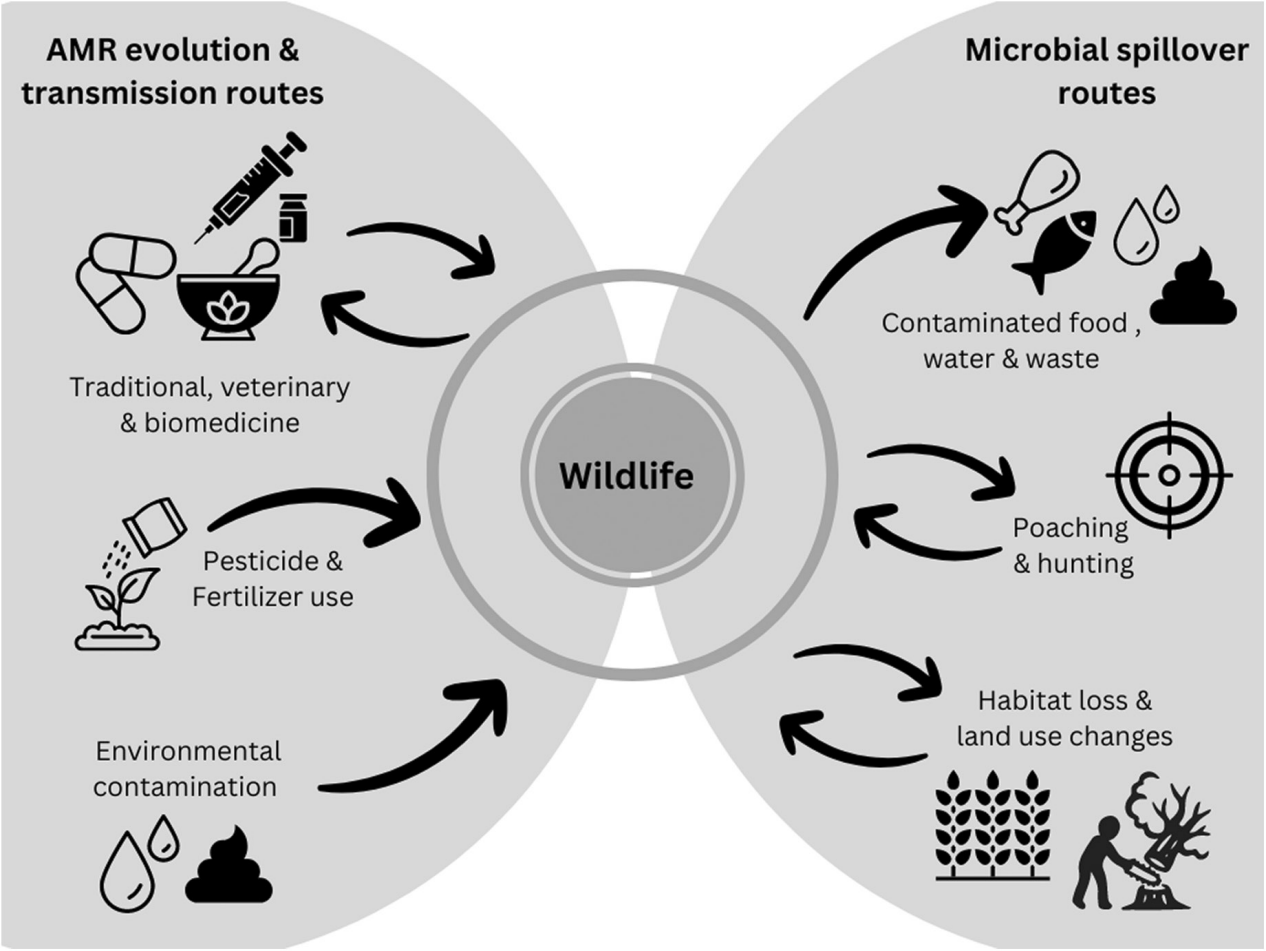
The many routes by which microbes and antimicrobials can move into, from, and through wildlife. As is evident in this graphic, most routes are created and acerbated by anthropogenic factors.

This absence of wildlife in AMR guidance seems counterintuitive, considering that recent disease outbreaks have demonstrated how influential wildlife is in the spread, divergence, and containment of One Health challenges (Otu et al., 2021; Shaheen, 2022). We are yet to determine the exact source of COVID‐19, but it is strongly suggested the virus originated in bats (Cui et al., 2023; Yang et al., 2023; Zhou et al., 2020) spreading to an intermediate host, the pangolin (Choo et al., 2020; Yang et al., 2023) before spilling over into humans via wet food markets (Nga et al., 2022; Wu et al., 2020). Pangolins are a globally exploited wildlife trade victim. After capture in the wild, they are often subject to poor hygiene and welfare conditions during captive breeding and transit to wet markets for live trade (Challender et al., 2019; Zhang et al., 2022). These situations facilitate incubation and spread of disease within species, plus routes of spillover into humans and domesticated animals once in captivity (Cui et al., 2023; Nga et al., 2022; Zhang et al., 2022). The presumed transmission pathway of COVID‐19 exemplifies how the exploitation and trade of wild species can provide pathways for novel and (re)emerging diseases to spill over into humans (Aguirre et al., 2020; Walsh et al., 2020). Similarly, national Ebola epidemics across African countries demonstrate links between HWC and One Health (Holmes, Dudas, et al., 2016; Judson et al., 2016; Leroy et al., 2004). Ebola viruses infect humans, primates, and some other mammal species with bushmeat consumption and forest encroachment for food, housing, and economic reasons being cited as the key drivers of Ebola spillover into humans (Judson et al., 2016). The consideration of wildlife and particularly how humans and domesticated animals interact with wildlife has been crucial to understanding the dynamics of such (re)emerging diseases (Otu et al., 2021). Applying a similar lens to AMR could thus have huge impact in understanding how mechanisms of resistance develop, how resistant genes and microbes spread, and how they impact on the health of our wider environments, including nondomestical species (Figure 1).

There is limited research into AMR evolution and spread within natural ecosystems (Ramey & Ahlstrom, 2020). Although recent studies have considered the impact of antimicrobial waste in global rivers, with some commentary on how this could contaminate natural settings, the primary focus is the contamination of water used for human and livestock consumption or agricultural irrigation (Grenni, 2022; Iossa & White, 2021; Reddy et al., 2022; Su et al., 2018). This again appears counterintuitive as the growth and migration of human populations globally, but especially within low‐and‐middle income countries (LMICs), means that humans, domestic and nondomestic animals can mix in a variety of environmental conditions from emerging cities, slums, small holdings, intensive farmland, industrial sites, and freshly disturbed natural habitats such as virgin forest (Connolly et al., 2021). Disease‐causing microbes, including those which are resistant, are likely to be moving between humans, livestock, domesticated and wild animals, and plants at a high rate (Amuasi et al., 2020; Bron et al., 2023; Cataldo et al., 2023; Connolly et al., 2021; Dolejska & Literak, 2019; Ramey & Ahlstrom, 2020; Shaheen, 2022). The consequences of this are relatively unknown but are likely to include increased exposure of all species to both novel microbes and the drivers of AMR. In combination, this may result in increased selection pressure for resistance (Cui et al., 2023; Dolejska & Literak, 2019; Palmeira et al., 2021; Ramey & Ahlstrom, 2020). What is clear is that wildlife is an unavoidable, but necessary, component within our One Health understanding of AMR (Figure 1). We cannot aim to develop meaningful action on AMR without considering how nondomesticated animals and natural environments impact, and are impacted by, resistant infections.

Wildlife and natural environments also contribute to drug development as many plants and microbes have antimicrobial properties (Cowan, 1999; El‐Saadony et al., 2023). Indigenous communities across the world use these in original or slightly processed forms (i.e., after cooking or combining with other products), while pharmacologists use natural compounds as a primary source of clinical antimicrobial discovery (Cowan, 1999; Weiner, 1980). Such natural antimicrobials may also allow wildlife to recover from infections without medical intervention. Indeed, several studies have demonstrated their health and growth promotion benefits in domesticated animals (Abou‐Kassem et al., 2021; Ogbuewu et al., 2023; Rafeeq et al., 2022, 2023) but there is no current comparable evidence for wildlife. Unfortunately, habitat degradation, climate change, pollution, intensive farming, and human encroachment are having a major influence on biodiversity including that of plants and microbes (Peixoto et al., 2022; Wang et al., 2018). A loss of biodiversity at any level has negative impacts on the functionality, productivity, and resilience of ecosystems (Hassell et al., 2023; Sutherland et al., 2023; Symstad et al., 1998; van der Plas, 2019). However, a loss of natural antimicrobial diversity could mean that wildlife has a limited arsenal of antimicrobial compounds in their natural environments, while also impacting pharmacological pipelines.

The challenges of pollution, habitat degradation, changes to farming practices, and human encroachment into natural habitats are all particularly acute issues within LMICs. As human populations expand and urbanize, there becomes an increasing demand for food and resources, plus greater generation of antimicrobial pollution from farms and hospitals. Couple this with an increased exposure to global AMR drivers such as climate change, and it seems feasible that wildlife could be at increased risk of health challenges and even death due to AMR. Additionally, if resistant genes and microbes can infect wildlife, it is unlikely to be long before they reach neighboring domestic animals, agricultural environments, and humans (Arnold et al., 2016; Cui et al., 2023; Greig et al., 2015; Ramey & Ahlstrom, 2020). An article published as this manuscript was under review provides empirical evidence that socioecological effects of urbanization can facilitate the transfer of mobile genetic elements between humans, livestock, and wildlife (Hassell et al., 2023) While this research focuses on zoonotic implications more generally, it adds weight to the argument that exploring AMR in nondomestic animals and their environment could be essential to support our understanding of the timeline, drivers, and consequences of resistance.

## 4 AMR AS AN EXAMPLE OF HUMAN–WILDLIFE CONFLICT (HWC)

As discussed, the spread and evolution of AMR can be rooted in HWC, including habitat degradation, intensive farming, pollution, and biodiversity loss. Thus, could the evolution and spread of AMR be considered a form of HWC in its own right? The International Union for the Conservation of Nature (IUCN) hosts a Human–Wildlife Conflict and Coexistence Specialist Group who consider HWC to be:

Struggles that emerge when the presence or behaviour of wildlife poses actual or perceived, direct and recurring threats to human interests or needs, leading to disagreements between groups of people and negative impacts on people and/or wildlife (IUCN SSC HWCTF, 2020).

The consideration of wildlife as a vehicle for the spread of AMR would certainly appear a “struggle” that could frame AMR as an example of HWC. Accepting this narrative would present critical questions to engage with on AMR (Table 1, Column 1).

**TABLE 1:** Critical research questions to engage with around the topic of AMR in wildlife and AMR as a form of HWC.

Research questions: AMR and HWC	Research questions: AMR and wildlife
What anthropogenic drivers of AMR impact upon wildlife?	Is wildlife health at risk from AMR infections?
What is the role of wildlife in the evolution and spread of AMR?	Could population decline, extinctions and biodiversity loss be linked to AMR?
Which forms of HWC are most likely to exacerbate AMR?	What would be the impacts of this loss of ecosystem functioning?
What HWC solutions could be used to minimize the evolution and spread of AMR?	What sources of antimicrobial exposure are wildlife at risk from and what are the health impacts of these exposures?

Over the past decade, a growing cohort of peer‐reviewed publications have touched upon these questions, particularly the role of wildlife in the spread of resistant genes and microbes (Dolejska & Literak, 2019; Palmeira et al., 2021; Torres et al., 2020). The 2016 review by Arnold et al. (2016) spearheaded recent research in this area after pointing out the data gaps regarding AMR in wildlife at molecular, organism, and ecosystem levels. However, many studies are species‐ or locally specific and do not consider One Health contexts beyond their focal system. This makes it challenging to determine the directionality of AMR, or to extrapolate findings to other species and ecosystems (Ramey & Ahlstrom, 2020; Swift et al., 2019). Additionally, conclusions and avenues for further research tend to frame wildlife as risk factors in the expansion of environmental AMR reservoirs and in the spread of specific resistant genes and microbes (Dolejska & Literak, 2019; Greig et al., 2015; Plaza‐Rodríguez et al., 2021; Torres et al., 2020). This narrative, framing wildlife as *risk factors*, is ultimately geared toward the prevention and spread of resistant infections to animal and environmental systems that are valuable to humans. Whether intentional or not, it is rooted within the ideology that human health is intrinsically worth more than that of other species. This is problematic as it places wildlife in the role of the villain, as is the case for many infectious agents (Kutz et al., 2009; Michel & Bengis, 2012). What is lacking in the literature is a consideration of AMR in terms of the intrinsic value of wildlife (Gomez et al., 2022).

There are limited data regarding the impact of resistant infections on nonhuman and nondomesticated species, which raises a suite of additional questions (Table 1, Column 2) around the impact of resistant infection on wildlife health. Framing AMR as a form of human–wildlife conflict could allow exploration of these pressing questions by pushing research directives to be informed by indicators of health beyond human and livestock surveillance. For example, health risks from exposure to pharmaceuticals have been extensively considered in birds due to severe declines in breeding success and population sizes of some endangered and threatened species. However, similar data are lacking for mammals, reptiles, and amphibians (Bean et al., 2022) and, to the best of our knowledge, there are no studies, which consider wildlife health in relation to antimicrobial exposure specifically.

## 5 HOW TO INCORPORATE WILDLIFE INTO THE CURRENT ONE HEALTH NARRATIVES ON AMR

Considering AMR as a form of HWC permits the utilization of currently untapped resources to address this critical One Health challenge. First, the knowledge of specialist actors currently missing from AMR research would have a route by which to share their expertise. These actors must include Indigenous communities who live alongside both wildlife, domesticated animals, and other human populations. Their working knowledge and experience of AMR through pollution, untreatable infections, and access to medications is likely to enrich the entire AMR landscape. However, with respect to wildlife specifically, Indigenous communities hold unique knowledge around changes in wildlife behavior, movements, feeding patterns, etc., that could be crucial to determining AMR drivers and health impacts. Additionally, zoologists, wildlife rangers, ecologists, veterinary wildlife professionals, social scientists, and the voluntary conservation team will be key players in bridging the AMR and HWC conflict knowledge gap. This multidimensional community is regularly engaged in HWC, wildlife health and conservation practice (Meredith et al., 2022). They are likely to be influential in supporting mapping of human‐wildlife‐livestock‐environment intersections and suggest ways of monitoring and minimizing the spread of AMR within and between populations.

Second, bringing AMR into the HWC space would facilitate data acquisition from a wider pool of species and environments. This may be biological surveillance data, qualitative interviews with practitioners and professionals, epidemiological data on disease, morbidity and mortality, or drug use data in conservation and veterinary sectors. All would be pivotal to better understand the evolution, spread, and impact of AMR in wildlife specifically and more broadly across the One Health sphere. Finally, HWC guidance could be used to mediate and improve AMR outcomes, including the spread of resistant infections within and between species and environments. For example, the recent IUCN guidelines on human–wildlife conflict and coexistence focus on the principles and processes that underpin multiple forms of HWC and suggest five key considerations, which facilitate sustainable coexistence. Such considerations mirror language used in many AMR guidance documents such as the country‐specific National Action Plans (NAPs), which discuss a need for awareness raising, cross‐disciplinary collaboration, data‐informed planning, and the development of long‐term solutions with political backing (WHO, 2015). As such, at country level it may be possible to develop wildlife‐aware AMR guidance that is not considerably different from existing National AMR Action Plans. It is rather a case of incorporating an additional perspective to nonhuman AMR drivers, surveillance, and health impacts.

In combination, the framing of AMR as a form of human–wildlife conflict and engaging with stakeholders and data in the HWC area will significantly enrich our understanding of AMR by accelerating transdisciplinary action to tackle AMR via a holistic One Health approach. It is becoming impossible to disregard the health implications of interactions between humans, wildlife and domesticated animals and environments (Hassell et al., 2023; Otu et al., 2021). However, current AMR narratives and global guidance neglect wildlife, and this represents a major gap in our understanding of AMR as a One Health challenge.

## AUTHOR CONTRIBUTIONS

**Jessica Mitchell:** Conceptualization (equal); data curation (equal); formal analysis (equal); funding acquisition (equal); investigation (equal); methodology (equal); project administration (equal).

## FUNDING INFORMATION

This work was funded by the University of Leeds via an internal Research Cultures Award to Dr Jessica Mitchell in January 2022.

## CONFLICT OF INTEREST STATEMENT

None to declare.

## Supporting information


Table S1

## Data Availability

NA.
