# Supplementary material for: Antimicrobial resistance (AMR) as a form of human–wildlife conflict: Why and how nondomesticated species should be incorporated into AMR guidance
Source: Ecol Evol. 2023 Aug 31;13(9):e10421. doi: 10.1002/ece3.10421 (PMC10468991; doi:10.1002/ece3.10421)
Supplement: Supplementary file 1 — Table S1 [file ECE3-13-e10421-s001.docx]

| **Global Guidance** | **Specialist Area Guidance** | **High Income Action Plans** | **Upper-middle income countries** | **Low and Lower-middle Income Action Plans** |
| --- | --- | --- | --- | --- |
| WHO Global Action Plan on AMR 2015  <https://www.who.int/antimicrobial-resistance/global-action-plan/en/> | Wellcome initiatives for environmental AMR (2019)  <https://wellcome.ac.uk/sites/default/files/antimicrobial-resistance-environment-report.pdf> | UK AMR 9 ambitions for change | 2016-2021 National Action Plan of the Islamic Republic of Iran for combating antimicrobial resistance | India action plan on AMR 2017 |
| IACG no time to waste 2019 recommendation  <https://www.who.int/antimicrobial-resistance/interagency-coordination-group/IACG_final_summary_EN.pdf?ua=1> | The European One Health Action plan against AMR (2017)  <https://ec.europa.eu/health/amr/sites/health/files/>  antimicrobial_resistance/docs/amr_2017_action-plan.pdf | Australian National action plan on AMR 2015 | National Action Plan on Antimicrobial Resistance, Costa Rica 2018-2025 | Cambodia national strategy to combat AMR 2015-17 |
| Flemming fund 2018-2022 research and development areas  <https://www.flemingfund.org/about-us/investment-areas/> | UK AMR strategy highlights 2019  <https://microbiologysociety.org/blog/highlights-from-the-uk-amr-strategy.html> | Strategic goals of Pan-Canadian framework for AMR action 2017 | South African AMR action plan 2018 - 2024 | Morocco National action plan on the control of AMR 2019 |
| The World Health Organization’s policy package to combat antimicrobial resistance  <https://www.who.int/bulletin/volumes/89/5/11-088435/en/> | CDDEP: Reducing antimicrobial use in food animals globally  <https://cddep.org/publications/reducing-antimicrobial-use-food-animals/> | Denmark National One Health Strategy against AMR 2017 | Fiji AMR action plan 2017 | National Action Plan for the Prevention and Containment of Antimicrobial Resistance, Kenya 2017 |
| GLASS surveillance objectives  <https://www.who.int/glass/en/> |  | Spain National action plan on AMR |  | National action plan on AMR Indonesia 2017-2019 |
|  |  | USA National Action Plan on AMR 2015 |  |  |
|  |  |  |  |  |

Table 1: Global and national AMR action plans screened for wildlife terminology between November 2022 and January 2023. National Action plans can all be accessed via <https://www.who.int/antimicrobial-resistance/national-action-plans/library/en/> links to additional documents are provided within the body of the table. This analysis was based on the screening methods of Mitchell et al 2022 (1) utilising the same dataset and rationale for document inclusion.

1. Mitchell J, O’Neill AJ, King R. Creating a framework to align antimicrobial resistance (AMR) research with the global guidance: a viewpoint. Journal of Antimicrobial Chemotherapy. 2022;77(9):2315-20.
